# Emerging Trends in the Remediation of Persistent Organic Pollutants Using Nanomaterials and Related Processes: A Review

**DOI:** 10.3390/nano12132148

**Published:** 2022-06-22

**Authors:** Salim Boulkhessaim, Amel Gacem, Samreen Heena Khan, Abdelfattah Amari, Virendra Kumar Yadav, Hamed N. Harharah, Abubakr M. Elkhaleefa, Krishna Kumar Yadav, Sami-ullah Rather, Hyun-Jo Ahn, Byong-Hun Jeon

**Affiliations:** 1 Department of Physics, Faculty of Sciences, University 20 Août 1955, 26 El Hadaiek, Skikda 21000, Algeria; salimbou21@yahoo.fr (S.B.); gacem_amel@yahoo.fr (A.G.); 2 Research & Development Centre, YNC Envis Pvt Ltd., New Delhi 110001, India; 3 Department of Chemical Engineering, College of Engineering, King Khalid University, Abha 61421, Saudi Arabia; abdelfattah.amari@enig.rnu.tn (A.A.); hhharharah@kku.edu.sa (H.N.H.); amelkhalee@kku.edu.sa (A.M.E.); 4 Department of Chemical Engineering and Processes, Research Laboratory of Processes, Energetics, Environment and Electrical Systems, National School of Engineers, Gabes University, Gabes 6072, Tunisia; 5 Department of Biosciences, School of Liberal Arts & Sciences, Mody University of Science and Technology, Lakshmangarh 332311, India; yadava94@gmail.com; 6 Faculty of Science and Technology, Madhyanchal Professional University, Ratibad, Bhopal 462044, India; envirokrishna@gmail.com; 7 Department of Chemical and Materials Engineering, King Abdulaziz University, P.O. Box 80204, Jeddah 21589, Saudi Arabia; rathersami@kau.edu.sa; 8 Department of Earth Resources and Environmental Engineering, Hanyang University, Seoul 04763, Korea; hjahn93@hanyang.ac.kr

**Keywords:** persistent organic pollutants, nanomaterials, degradation, remediation

## Abstract

Persistent organic pollutants (POPs) have become a major global concern due to their large amount of utilization every year and their calcitrant nature. Due to their continuous utilization and calcitrant nature, it has led to several environmental hazards. The conventional approaches are expensive, less efficient, laborious, time-consuming, and expensive. Therefore, here in this review the authors suggest the shortcomings of conventional techniques by using nanoparticles and nanotechnology. Nanotechnology has shown immense potential for the remediation of such POPs within a short period of time with high efficiency. The present review highlights the use of nanoremediation technologies for the removal of POPs with a special focus on nanocatalysis, nanofiltration, and nanoadsorption processes. Nanoparticles such as clays, zinc oxide, iron oxide, aluminum oxide, and their composites have been used widely for the efficient remediation of POPs. Moreover, filtrations such as nanofiltration and ultrafiltration have also shown interest in the remediation of POPs from wastewater. From several pieces of literature, it has been found that nano-based techniques have shown complete removal of POPs from wastewater in comparison to conventional methods, but the cost is one of the major issues when it comes to nano- and ultrafiltration. Future research in nano-based techniques for POP remediation will solve the cost issue and will make it one of the most widely accepted and available techniques. Nano-based processes provide a sustainable solution to the problem of POPs.

## 1. Introduction

Over the past few decades, water pollution containing organic pollutants has seen a tremendous increase due to the high consumption of such pollutants. Persistent organic pollutants (POPs) are a group of hazardous chemical compounds that originate from anthropogenic activities during production, utilization, and disposal [1]. They can impact living beings and the environment adversely because of their ease of transportation by wind and water. The level of hazardous persistent organic pollutants is increasing every day in the environment. The appearance of POPs in groundwater and drinking water is a significant threat as they can be easily transported from one source to other and released into a new environment. POPs remain in the environment for a prolonged period and have the potential to accumulate in the body, and they are found to be extremely toxic even at the lowest concentration [2]. They can easily pass from one organism to another via the food chain, also affecting the organisms far from the site of contamination. Due to their persistent nature, long-range transportability, and tendency to bioaccumulate, POPs became a global concern [3]. The Stockholm Convention classified 22 nonpolar organic molecules as POPs. The Organochlorines (OCs) comprise the largest class of POPs, including aldrin and dichlorodiphenyltrichloroethane (DDT), followed by organophosphorus (OPs) pesticides, then by flame retardants and polyaromatic hydrocarbons (PAHs) and other chemicals (polychlorinated biphenyls PCBs); the byproducts of the above-mentioned compounds also constitute for the same [4,5].

The release of POPs in water bodies is an issue of concern given the environment and human safety due to their tendency to become bioaccumulated and biomagnified throughout the food chain (FC). POPs may gain excess to the body of human beings via drinking water through the leaching of pollutants [6]. It is difficult to implement and develop advanced technology for the remediation of such pollutants, which is very efficient as well as cost-effective with low maintenance. Among various wastewater-treatment technologies, advancement in nanotechnology has been found attractive by researchers across the globe The present review highlights the significant developments in nanotechnology concerning the treatment of POPs, as nanotechnology has emerged as a promising and innovative technology and novel nanomaterials can be utilized for the efficient degradation of such pollutants. The present review intends to highlight the current research in the use of nanotechnology for the removal of POPs and also focus on the impact of POPs on the environment. The review aims to provide a critical analysis of various articles published on the utilization of nanomaterials for the remediation of POPs from contaminated waters, so that it further contributes to the development of novel nanomaterials for such applications.

## 2. POPs, Source, and Fate

POPs have grabbed significant attention worldwide. POPs are defined as xenobiotic chemical compounds of different origins, but all have similar characteristics, i.e., high toxicity, bioaccumulation, hydrophobicity, environmental persistence, and ability to transfer via the FC [7]. POPs are carbon-containing chemicals, and due to their higher solubility in the lipids, they tend to become accumulated among the fatty tissues and can disrupt the endocrine system of organisms, therefore often referred to as endocrine disruptors (EDs) [8]. The physicochemical properties of POPs are responsible for their dispersion and distribution in the environment; POPs have low water solubility (log Kow 3–7); therefore, they have high adsorption, low degradation, and hydrophobic nature [9].

### Various Categories of POPs

**Figure 1:**
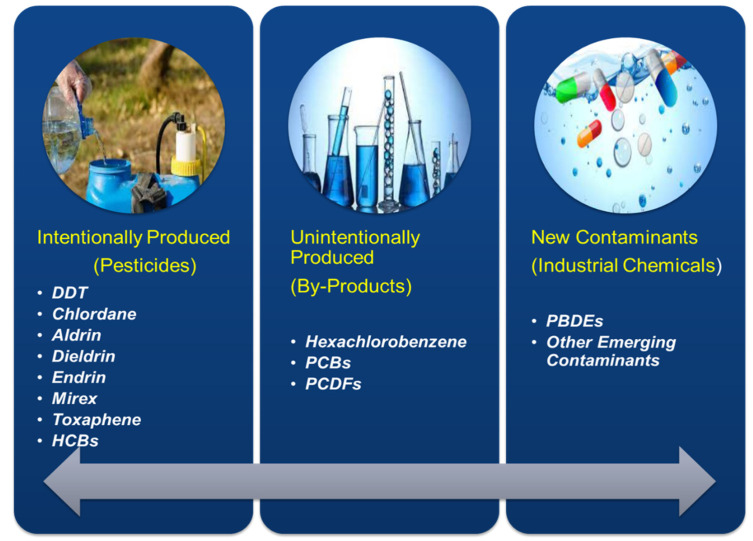
Type and categories of POPs.

The utilization of POPs was restricted since 1970 in various parts of the United States of America (USA) and Europe. Moreover, there was a strict prohibition on the release of such POPs in both the above-mentioned continents [10]. The use and consumption of pesticides increased abruptly after the green revolution; at that time, the hazardous effects of POPs were unknown. Lately, the toxic effects of pesticides have emerged globally. The general public started to understand the toxicity of pesticides and other organic contaminants. After the Stockholm convention, POP was placed into three categories, i.e., pesticides, by-products, and industrial chemicals [11]. Figure 1 depicts the type and different categories of POPs.

POPs are the range of synthetic hazardous chemicals, produced either intentionally or unintentionally [12]. Pesticides fall under the category of the intentionally produced chemicals used to control pests in agriculture and houses; DDT is a known such example that was banned globally due to its extreme toxicity [13]. Others are industrial products or unintentionally produced chemicals, i.e., dioxins. The new class of POPs includes types of emerging contaminants such as polybrominated diphenyl ethers (PBDE), perfluorinated compounds (PF), and a list of new contaminants added day by day.

POPs can be classified as Organochlorine Pesticides (OCP); hexachlorobenzene (HCB) and other polychlorinated benzenes (PCBzs); PAHs; polychlorinated naphthalenes; PCBs; polychlorinated dibenzo-p-dioxins and dibenzofurans (PCDD and PCDF); and other contaminants of emerging concerns [14]. At first, during the Stockholm convention (2001), the participating countries decided to minimize or strike out the production, usage, and release of 12 key POPs popularly referred to as “the dirty dozen”. Later, ten more chemical substances were added to the group of POPs after two amendments (2009 and 2011) [15]. Table 1 summarizes the list of twenty-two POPs after the Stockholm Convention.

POPs can sustain in the environment for prolonged periods, taking decades or several centuries to be completely degraded. Due to their physicochemical properties, POPs have the tendency to travel long distances and resist degradation (biological and chemical degradation), which allows them to bioaccumulate to a deeper level via biomagnification, and their exposure can lead to severe damage to health and the environment [16]. Several studies suggest a range of adverse effects induced by POPs, as most of them are semivolatile compounds and can easily absorb onto the atmospheric particles and migrate into the water, air, and soil media [17,18,19]. POPs are rarely found in one environmental medium but are present in all media, and if tested will be found to be present in all media across the world [20]. POPs are found in agricultural wastes, chemical, and electronic industry waste, as well as pharmaceutical waste.

POPs are severely toxic that even the smallest concentration of them is found to be highly fatal to the organisms. POPs are generally resistant to chemical, biological, and photodegradation as they have low solubility and it is quite difficult to degrade POPs using traditional wastewater-treatment technology [21,22]. In the recent past, remediation of POPs was achieved by advanced wastewater-treatment technologies or by the combination of one or two methods. However, the most important question arises: Despite all technologies, why are POPs resistant to most degradation processes? POPs generally exhibit lipid solubility, and because of this reason, they tend to accumulate in fatty tissues of organisms. Moreover, halogenated compounds show great stability toward hydrolysis and photolytic degradation due to the nonreactivity of c-cl bonds [23]. The stability towards degradation and lipophilicity of POPS makes them compounds of particular concern. POPs are also divided into four levels based on their toxicity:

(i) most hazardous chemical [restricted for production and utilization];
(ii) medium level chemicals [confined to use during the production];
(ii) unintentional discharge of chemical;
(iv) use of chemicals under investigation.

As we know, POPs are extremely toxic halogenated compounds that largely impact humans either through point or non-point sources. The organic pollutants generally consist of personal care products (PCP), pesticides, organic dyes, endocrine disruptors, pharmaceutical waste, and other such contaminants of emerging concern [24]. The release of POPs in water bodies causes disturbance to the aquatic food chain, as POPs tend to bioaccumulate, and EPA indicated that the rate of disease caused by POPs is very high in coastal and marine ecosystems [25]. Thus, POPs tend to impact every living organism in some or the other way due to their hazardous nature. There arises the need for the remediation of such pollutants from the environment by applying advanced techniques, but the results show that the conventional technologies were not efficient for the complete removal of POPs as they are simply transforming the pollutants from one phase to another rather than the complete elimination [26]. With the advancement of nanotechnology for environment application, the focus has been shifted to the removal of POPs using nanomaterials. The present review significantly highlights the utilization of nanotechnology for the removal of POPs from the environment.

## 3. Conventional Treatment Technology

It is well-proven from the literature that there is a drastic decrease in the utilization of conventional methods for wastewater treatment due to the presence of POPs and other emerging pollutants. The existing treatment methods for instance oxidation, activated carbon, activated sludge, and reverse osmosis are not efficient to treat the range of POPs that are highly toxic, and the by-product produced during the process of degradation can prove to be more hazardous than the parent compound [27]. Therefore, there should be effective treatment technology for the treatment of POPs where there is no need for secondary or tertiary treatment.

**Figure 2:**
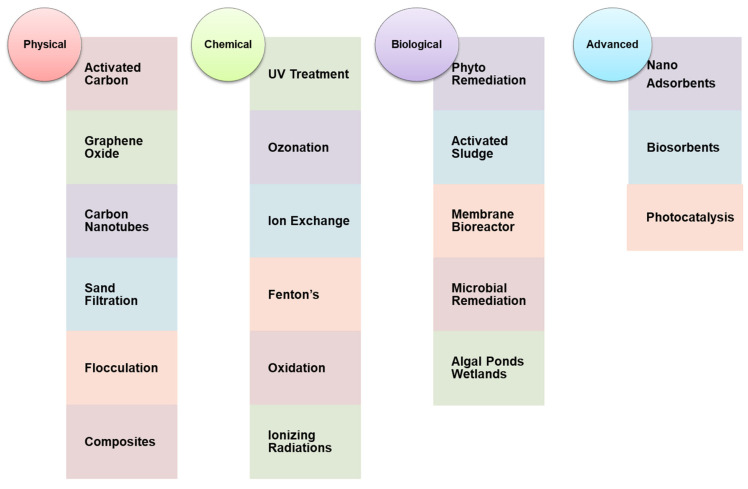
Conventional treatment technologies.

Given the Stockholm convention, very slow progress has been observed in the destruction of POP; several research studies showed the presence of a higher percentage of organic contaminants in the water bodies of those countries where the application of POPs has been confined [28]. The conventional approaches leave pollutants for the coming generations, and it does not go sustainably. In order to surpass the demerits of traditional treatment technologies, the advanced alternative method should be sought to completely degrade the POPs into environmentally friendly or less toxic end-products. Figure 2 shows the list of various conventional treatment technology used for the remediation of POPs.

As per Figure 2, every wastewater treatment involves physical, biological, and chemical methods, which occur in three basic stages, i.e., primary, secondary, and tertiary treatment, followed by advanced treatment technology [29]. Such methods are used for simple water purification such as filtration, disinfection, decoloration, sedimentation, coagulation and flocculation, steam distillation, ion exchange, deionization, reverse osmosis, and biological treatment using microorganisms [30]. Materials used in traditional technologies are quite costly, and there are chances to give rise to secondary pollutants. The requirement for advanced and efficient methods of removal of POPs from waters and wastewaters results from various studies that show the incompetence of routinely used conventional methods of treatment. Nanotechnology allows an exceptionally efficient, sustainable, eco-friendly, and sustainable process that can provide a promising alternative to conventional technologies with high performance and low-cost solutions [31]. Table 2 shows the advantage of widely used conventional techniques and advanced nanotechnology-based processes for the removal of organic pollutants.

Based on the discussion above, conventional treatment technologies can treat most of the organic pollutants to a certain extent, but to overcome the drawbacks associated, it is necessary to look for efficient advanced treatment technologies considering the nature of pollutants and other environmental factors. The alternative treatment technology should be effective and sustainable and provide a single-step solution to the existing problem without generating secondary pollutants.

## 4. Nanotechnology for Environment Remediation

In the present era, no one is unaware of nanotechnology and its application. Nanotechnology generally deals with the particle at the nanoscale level (1–100 nm). Nanoparticles are particles with at least one dimension in the nanoscale, that have unique physicochemical properties, high surface–volume ratio (SVR), and exceptional optical and electrical properties, making them potential candidates for advanced applications in electronics, medicine, and the environment [32]. For now, nanotechnology arises as the most cutting-edge technology for effective wastewater treatment and other hazardous contaminants in a sustainable manner. Nanomaterials can be used for the remediation process based on their nature, such as (i) nanocatalyst, (ii) nanoadsorbents, and (iii) nanomembrane; these three classes are widely used in the treatment of wastewater [33]. Due to their smaller size, nanoparticles have a tremendously higher surface area, which increases the surface chemical phenomenon of nanoparticles, allowing the chemical reaction over their surface [34]. Nano-based processes are highly efficient and multifunctional and require less reaction time, also providing an affordable solution to wastewater treatments as compared to other conventional methods. The nanomaterials used for the remediation of POPs can be classified as metallic (i.e., metal/metal oxides; carbon-based nanomaterial, nanocomposites) and nonmetallic (i.e., Nanomembranes, polymeric nanomaterials, metal-organic frameworks, etc.) [35]. Oxidations and adsorption of organic pollutants followed by degradation are the most common strategies for the nano-based remediation of organic pollutants [36]. Table 3 summarizes the reviews published in the field of treatment of POPs using different nano-based processes during the last five years.

## 5. Advanced Nanotechnological Approaches for Removal of POPs

The fate, transportation, and degradation of POPs involves complex reactions, and to successfully remove POPs, economic and technological factors are critically considered before selecting the appropriate treatment strategy, as they can highly impact the removal of POPs. Nanotechnology emerges as the biggest blessing for the environmental problems as one of the most promising approaches to transforming contaminated treatment technologies. More simply, nanoremediation can be defined as the use of nanomaterial for the treatment of environmental contaminants; nanoremediation is an innovative approach to the sustainable removal of organic pollutants such as pesticides, PCBs, chlorinated solvents, heavy metals, PCBs, brominated chemicals, and other hazardous compounds [57,58]. The unique optical, thermal, mechanical, structural, and electromagnetic properties of nanomaterials make them beneficial for various possible applications. Nanomaterials can be synthesized using either physical, chemical, or biological approaches and as nanoparticles, nanoadsorbents, nanosensors, nanocatalysts, or nanomembranes for wastewater treatment [59]. Nowadays, green-chemistry-based techniques are extensively applied for the synthesis of nanomaterials for environmental applications, providing a sustainable alternative to chemical-free methods with zero-waste generation [60,61]. Table 4 summarizes the various nanomaterials applied for the removal of POPs.

**Figure 3:**
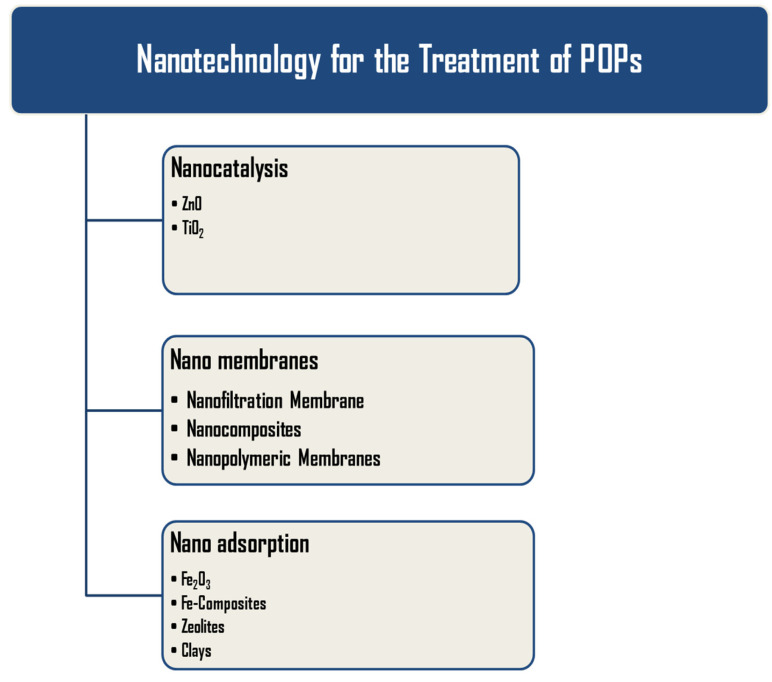
Nanotechnological processes used for the treatment of POPs.

Moreover, several studies support the synthesis of advanced multifunctional nanomaterials such as nanowires, nanoflowers, nanorods, nanocomposites, etc., to enhance the efficiency of nanomaterials to overcome the current challenges [98]. The higher SVR of nanomaterials enhances the surface reactivity with the POPs and provides a high reaction area over their surface [99]. In the present section, advanced nanotechnological approaches will be discussed, which are used for the treatment of POPs in the following sections, nanocatalysis; nanoadsorbents, and nanomembranes. Figure 3 depicts various nanotechnological approaches used for the removal of POPs.

Gibbs free energy ΔG is the amount of energy available for any chemical reaction to occur, it plays a significant role in determining the fate of POPs transformation into different intermediate and end products. It decides the fate of the rection whether the degradation process is spontaneous or nonspontaneous; by using ΔG values one can determine the amount of energy liberated from any biochemical reaction [100]. It is a known fact that POPs are resistant to most of the environmental-degradation process, but still, some molecular alteration could be possible that leads to the formation of an even more toxic intermediate- or end-product. In the later sections, the use of nanomaterials for the treatment of POPs under different headings will be studied systematically.

### 5.1. Nanocatalysis

**Figure 4:**
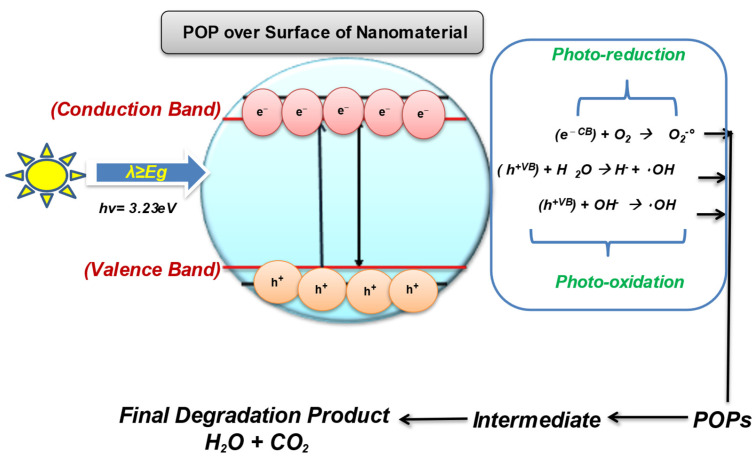
Photocatalysis over the surface of the nanomaterial.

With the ineffectiveness of conventional technologies to completely degrade and mineralize the organic pollutants, there arises the need to develop a green, innovative, and sustainable method that can destroy the POPs with much less energy consumption and chemical utilization [101,102,103]. Therefore, the scientific community has started looking for advanced oxidation processes as a low-cost and effective method that is proficient in oxidizing and mineralizing a range of pollutants, including POPs, due to their strong oxidizing radicals [104]. The use of semiconducting wide-bandgap nanomaterials for the treatment of contaminants into eco-friendly compounds comes under nanocatalysis. The semiconductor metal and metal-oxide nanomaterials have gained significant attention in POPs treatment sustainably. Several types of nanocatalyst are used for the effective degradation of POPs from wastewaters such as Fenton-based catalyst, electrocatalyst, photocatalyst, and even doped multifunctional nanocatalyst [105,106]. Photocatalysis/nanocatalysis is a well-known AOP; it is used to enhance the biodegradability of POPs by using oxidants to degrade organic pollutants by the release of highly reactive oxygen species (ROS) for the chemical reaction to occur [107]. Photocatalysis involves the catalytic activation in the presence of light and relies on the generation of strong radicals, i.e., H_2_O_2_, O_2_ ^•–^, O_3,_ and OH radicals, which destroy almost all organic molecules [108]. Photocatalysis is even effective for the remediation of volatile organic compounds (VOCs) such as PCBs, Dioxins, and PHA by producing free radicals. The process of photocatalysis starts as the nanocatalyst with a wide bandgap (such as ZnO, TiO_2_, WO_3_) becomes photoexcited in the presence of a light source (natural or artificial) and oxygen used to degrade POPs [109,110]. The photocatalytic-degradation process ideally involves the following steps, as shown in Figure 4.

As of now, TIO_2_ and ZnO are the most widely utilized semiconductors for the degradation of POPs. The heterogeneous photocatalyst is efficient for removing highly hydrophobic POPs from the environment. The surface, pore-volume, and structure of the semiconducting material are deciding parameters to consider while selecting the ideal catalyst [111,112]. The surface properties and crystal structure of the material can be tuned to boost the degradation efficiency of the photocatalyst [113]. However, the main demerits related to photocatalysis is the removal of nanomaterial from the reaction media once the process is over.

Lwin et al. (2019) synthesized a cubic ZnO-SnO_2_ nanocomposite via the solvothermal method and used it for the degradation of tetracycline hydrochloride by photocatalysis. The as-synthesized nanocomposite material was analyzed by advanced instrumentation. The degradation result shows that ZnO-SnO_2_ nanocomposite shows remarkable photostability even after four consecutive cycles, providing a successful method for the remediation of POPs that can also be used for the remediation of other organic contaminants [114].

Amir et al. (2016) reported a MnFe_2_O_4_@PANI@Ag nanocatalyst to degrade azo dyes from industrial waste. The degradation result proves that as-synthesized nanocatalyst has a high potential to degrade the azo dyes, and the best advantage is that the nanocomposites can be easily detached by applying the external magnet and can be used for the next cycle with the same efficiency [115].

Khan et al. (2018) synthesized magnetic Fe-ZnO nanocomposite material via the sonochemical process to remediate Chlorpyrifos pesticide from the aqueous solution. The result shows that Fe-ZnO nanocomposite was quick to degrade the pesticide with good stability and reusability. The results show up to 90% degradation and the nanocomposite could be removed by applying the external magnet [73].

Chen et al. (2022) explored Mn-based nanocomposites to degrade bisphenol A, as the potential of pristine manganese oxides (Mn_3_O_4_) for the remediation of organic substances has not been explored yet. The study involves the activation of Mn_3_O_4_-based peroxymonsulfate to degrade bisphenol A (BPA) in different water systems. The results show remarkable mineralization (75.9%) with efficient removal of BPA (96.7%) at optimum parameters in 60 min. The nanocomposite shows the stability of Mn_3_O_4,_ long-term performance, and eight cycles of reusability with only an 11% reduction in BPA removal. The activated-sludge inhibition method used to check the toxicity of BPA after degradation was shown to be significantly repressed [116].

Photocatalysis is undoubtedly the most widely employed process for wastewater treatment. High efficiency, sustainability, and good results make photocatalysis a method of choice for the degradation of a wide range of organic and inorganic pollutants.

### 5.2. Nanoadsorption

Nanoadsorbents provide high sorption efficiency because of their extremely large surface area and sorption sites, tunable pore size, much lower intraparticle-diffusion distance, and high surface activity for effective adsorption of a vast range of organic and other pollutants [117,118,119]. The advantage of using nanoadsorbents is that they can be easily functionalized to make them highly selective for any pollutants [120]. The adsorption process has been found to be successful for the remediation of POPs such as hydrocarbon, dyes, phenols, detergents, pharmaceuticals, pesticides, and biphenyls.

**Figure 5:**
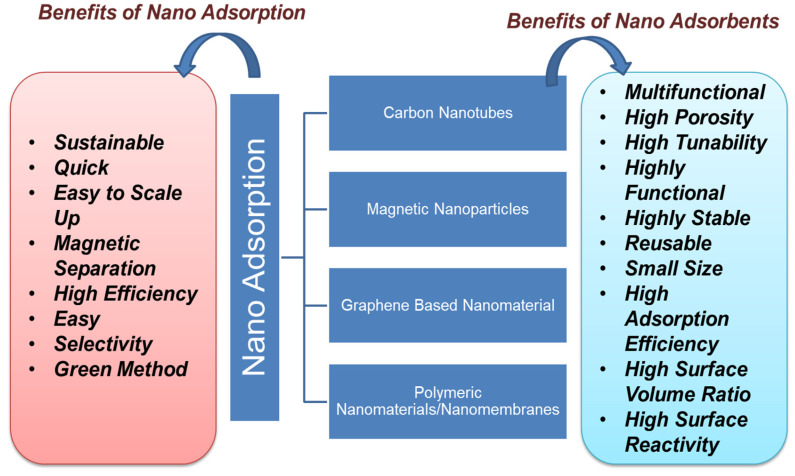
Types and benefits of nanoadsorption.

Figure 5 shows the types of carbon-based nanoadsorbent material with their benefits. Nanoadsorption is an easy and safe process for the remediation of POPs from water bodies. Among various technologies, nanoadsorption so far emerges as a widely efficient method for the remediation of POP. Several studies prove the efficiency of nanomaterials for the adsorption of various POPs from the wastewater, as more than 90% removal efficiency was achieved in most of the studies for up to ten cycles [121]. The adsorption efficiency of nanomaterial is mainly monitored by producing a complex with the surface of metal oxides and enduring a one-electron oxidation reaction under visible irradiation. Nanoadsorption is based on electrostatic interactions, hydrogen bonding, and hydrophobic interactions such as van der Waals, electron donor–acceptor, etc. [122]. Nanomaterials such as clay, zeolite, alumina, metal/metal oxides, activated carbon, carbon-based nanomaterials, nanocomposites, nanosheets, nanotubes, chitosan-based polymers, and graphene-based nanomaterials are extensively applied in the process of nanoadsorption [123]. For effective removal of POPs, the use of magnetic nanoparticles, especially iron oxide, has led away to a new class of magnetic-separation strategies. Microporous structures present in activated carbon aid the adsorption efficiency in the removal of POPs [124,125]. Carbon-based nanoadsorbents tend to interact with contaminants due to hydrophobicity, hydrogen bonding, and covalent and electrostatic interactions [126]. Each form has several adsorption sites that can absorb the organic pollutants due to their flexibility. Both single-walled and multiwalled carbon nanotubes have been surface-modified by increasing the porosity to generate high-energy sites to adsorb more organic pollutants over increasing the efficiency of manifolds [127,128].

A study was conducted by Ali et al. (2018) for the adsorption of cyanazine using green-synthesized iron nanocomposites. The result shows the quick removal of cyanazine from water due to low contact time [129]. In another study by Mahdavi et al. (2021), aminoguanidine-modified magnetic graphene oxide was used for the efficient remediation of chlorpyrifos from water. The desorption of chlorpyrifos was analyzed by HPLC-MS, and the results show remarkable desorption by using a synthesized nanoadsorbent [130].

Izanloo et al. (2019) successfully synthesized bifunctional nanoadsorbent (Fe_3_O_4_@SiO_2_@NH_2_@SH) for the remediation of 2,4-dichlorophenoxyacetic acid (2,4-D) and lead from synthetic wastewater. The adsorption process follows the Langmuir isotherm with second-order kinetics. Based on the study, it was noticed that pH plays a key role in the adsorption of organic contaminants. The result also shows that the synthesized nanoadsorbent was so efficient that it can be used for several cycles without losing its desorption efficiency [131].

Mohammadi et al. (2018) modified magnetic Fe_3_O_4_@SiO_2_@NH_2_ nanoadsorbent for the remediation of 2,4-Dichlorophenoxyacetic acid (2,4-D) and 2-methyl-4-chlorophenoxyacetic acid (MCPA) from aqueous solution. The effects of pH, time, dosage, and initial concentration of the pollutants were studied to obtain a better insight into the synthesized material. The result shows that amino-functionalized Fe_3_O_4_@SiO_2_@NH_2_ is an effective adsorbent for the remediation of phenoxy-acid herbicides from water due to its advantages such as easy and rapid separation of the target pollutant from the solution [132].

Dehghani et al. (2019) investigated the adsorption of diazinon on multiwalled CNTs in a batch reactor. The results show that 100% remediation of diazinon was achieved at pH 6 in just 15 min. The result shows that the highly efficient MWCNTs can be used for the remediation of different pesticides from an aqueous solution [133].

Kalhor et al. (2018) synthesized amino-functionalized nanosilica (NH_2_-SHNS) nanoadsorbent for removal of imidacloprid pesticide from wastewater. The as-synthesized nanoadsorbent had a spherical shape in the size range of 70–250 nm. Parameters such as pH, temperature, dosage, and concentration of pesticide were investigated and observed that the adsorption equilibrium was matched with the Redlich–Peterson isotherm and follows pseudo-first-order reaction kinetics [134].

Sahoo et al. (2020) synthesized magnetically separable GO/g-C_3_N_4_-Fe_3_O_4_ nanocomposite using the hydrothermal process for the remediation of methylene blue and tetracycline from wastewater. The result exhibited that nanoadsorbents can be used for up to 5 cycles without losing their efficiency. It was observed that the adsorption of pollutants is pH-dependent, and maximum adsorption capacity was achieved at pH 3 for tetracycline and pH 9 for methylene blue. The higher adsorption efficiency is due to hydrogen bonding and π-π interaction. The adsorption data follow pseudo-second-order kinetics and are best-fitted to the Langmuir isotherm [135].

Nikzad et al. (2019) studied the adsorption of diazinon by magnetic guar-gum MMT (montmorillonite) from aqueous solutions. The magnetic MMT was synthesized via the chemical coprecipitation method and was found in a size range of 50–130 nm. The adsorption kinetics follows the pseudo-second-order model, also best following the Langmuir isotherm. The magnetic MMT shows excellent adsorption efficiency for the removal of diazinon [136].

In a similar study, Peralta et al. (2020) synthesized silica-based nanoadsorbents for the remediation of several POPs. The hybrid magnetic iron-oxide nanoparticles were covered with silica and 3-(trimethoxysilyl) propyl-octadecyl dimethyl-ammonium chloride; it is further modified to obtain the final nanoadsorbent [137].

The process of nanoadsorbents is widely used as a low-cost, effective, and sustainable treatment technology. It is most successful for the removal of heavy metals from wastewater due to its reusable efficiency and it does not require high operation and maintenance costs. Magnetic nanoadsorbents are easy to separate from the reaction medium by applying the external magnet, which is one of the greatest advantages of using nanoadsorbents.

### 5.3. Nanofiltration

Nanotechnology has paved the way to advance water treatment systems by using nanofiltration membranes [138]. Membrane processes such as microfiltration (MF), reverse osmosis (RO), ultrafiltration (UF), and nanofiltration (NF) are pressure-driven filtration techniques and are considered highly effective processes for the treatment of wastewaters [139]. They are considered alternative methods for the remediation of organic micropollutants for the water bodies. Though the treatment of wastewaters using membrane processes is costly, they are the best alternative to conventional techniques as their removal efficiency is very high [140]. The nanoparticle can be incorporated into the membranes either by surface immobilization, blending, or surface grafting for developing the membranes with desirable functionality and characteristics [141]. By using the electrospinning method, polymeric or composite nanofibrous membranes can be developed to compose ultrafine nanofibers by using materials such as ceramics, biomass wastes, polymers, or metals in the range of 10–1000 nm [142,143].

Out of all membrane techniques, NF and RO proved their efficiency for the effective filtration for the remediation of micro/trace organic pollutants. NF is comparatively more efficient for the remediation of pollutants than RO (a drawback of high energy consumption and maintenance cost), where filtration is caused by different mechanisms, i.e., convection, diffusion, and charge effects [144,145,146]. NF is effective for the remediation of micropollutants due to its small pore sizes, high efficiency, and user-friendliness [147]. Several polymers (natural and synthetic) have been used for the preparation of nanofiltration membranes such as polypropylene, polyvinyl fluoride, polyacrylonitrile, and most commonly cellulose acetate, as they are effective in the removal of POPs [148,149,150,151]. Nanofibers have stable adsorption structures due to their loose bundles as compared to nanotubes and nanoparticles. Nanofibers have been found to be efficient for the removal of pesticides from wastewaters through their molecular propagation mechanism; furthermore, when semiconducting materials are used for the synthesis of nanofibers, they can add the photocatalytic property [152]. Several nanocomposite nanofiber (ZnO-cellulose acetate, TiO_2_-graphene, etc.) membranes exhibit strong photocatalytic efficiency for the remediation of dye compounds [153]. In addition, the immobilization of magnetic nanoparticles with the membrane was found efficient for the remediation of organic pollutants, and doping with TiO_2_ for photocatalytic degradation shows good results [154].

For the quality and efficient removal or range of organic/inorganic pollutants, single or combinations of filtration techniques (i.e., ultrafiltration; microfiltration; nanofiltration, and a combination of two or more) have been utilized. Moreover, a combination of filtration techniques with biological or chemical methods is known for the efficient remediation of persistent organic pollutants from wastewaters [155]. However, for the successful implementation of membrane processes, the following factors need to be considered: type of membrane, membrane modules, membrane composition, and most importantly membrane interaction with the pollutant [156].

Nanofiltration is a pressure-driven technique based on hydrodynamics between the membrane surface and membrane nanopores and is efficient in the remediation of low-molecular-weight compounds with a size range between 1–10 nm [157]. By reducing the hardness of organic pollutants, nanofilters help to reduce the ionic strength of the solution. The effectiveness of filtration is vastly dependent on the surface concentration of the membrane, its porosity, and charge [158]. The electrospinning technique is used for the preparation of high-quality nanofibrous membranes [159]. Nanofiltration effectively removes almost all dissolved salts and rejects multi, di- and univalent ions, so it is highly efficient for the treatment of arsenic in drinking water [160].

The study was conducted by Karimi et al. (2016) for the effective removal of atrazine and diazinon from wastewater by using a thin-film composite polyamide nanofiltration membrane synthesized via interfacial polymerization. The results show that diazinon was better rejected than atrazine. The water permeability and diazinon rejection increased from 22 L/m^2^/h and 95.2% for the unmodified membrane to about 41.56 L/m^2^/h and 98.8% for the 2% (*w*/*v*) TEA modified membrane showing a significant improvement in the performance of poly (piperazine amide) TFC NF membranes for pesticides removal [161].

Wang et al. (2020) synthesized a novel nanocomposite with catalytic property (Al-MOF/Fe_3_O_4_/PDA@Ag) by loading silver nanoparticles (Ag) onto the magnetic Al-MOF/Fe_3_O_4_/PDA. The as-synthesized composite shows higher removal efficiency for various organic pollutants (CIP, NOR, and MO) in a short period. The catalyst could be easily separated by the application of an external magnet and also shows good reusability and stability [162].

Membrane filtration is found to be the safest technology, and NF is excellent for the removal of low-molecular-weight compounds. NF is the only filtration technology known for the removal of pesticides and other organic contaminants successfully. However, membrane blockage and fouling are the drawback of filtration technology, which can be overcome by the use of hybrid technologies.

### 5.4. Nanobiotechnology and Hybrid Technologies

Nanobiotechnology emerges as a green and sustainable alternative for the removal of POPs through the process of bioremediation and applications of nanoremediation. Nanobioremediation is the process where microorganisms along with nanoparticles are used to remove the pollutants; it is of different types, i.e., phytonanoremediation (plant + NP) and micronanoremediation (microbes + NP) [163]. The nanoremediation process is mostly dependent on the sorption process and involves both adsorption and absorption. Thermodynamic, mechanistic, and kinetic studies are essential to understanding the behavior of nanomaterial and contaminant interaction. Even various nanomaterial is used to increase the efficiency of microbial degradation.

As discussed in the earlier section, the process of bioremediation is lengthy and less effective. Therefore, the combination of bioremediation and nanoremediation makes it highly effective and efficient. The process of bioremediation is based on the biotransformation of the target pollutant through catabolic enzymes to the end product, and the efficiency of remediation is based on the microorganisms, property of the pollutants, and the environmental factors; hence, the focus has been shifted towards the hybrid technologies, which involve the combination of two or more different technologies for the effective removal of environmental pollutants. The most effective hybrid technology is a combination of photocatalysis with membrane filtration; techniques of nanobiotechnology also pave their way towards the one-step removal of a wide range of organic pollutants [164]. Along with the novel nanomaterials, further, there is a need to develop integrated or hybrid nanomaterials to improve remediation processes, as we know that each technology has its own sets of merits and demerits and a specific range of removal efficiency. Nanoadsorbent materials have exceptional properties for the remediation of an extensive range of heavy metals whereas nanocatalysts can effectively degrade the POPs into eco-friendly compounds, both in a sustainable manner.

## 6. Case Studies from Different Countries

Over the past years, several technologies have been demonstrated for the effective removal of POPs for the environment in different countries such as the USA, Canada, Japan, China, Australia, etc. Even the United Nations Environment Programme (UNEP) recognizes some of the methods for the removal of PCBs. Photocatalytic degradation reaction is currently the most common yet effective method used for the removal of POPs from water and wastewaters; however, the adsorption process is used for the removal of POPs from the marine environment.

## 7. Future Prospects

To ensure a sustainable future, there is a significant need of advance treatment technology for POPs. The treatment of POPs has failed to achieve 100% success using conventional technologies because they are inefficient to reach a zero-elimination rate. As per several reports published on the removal of POPs, most of the complications faced during the treatment of POPs have been solved by successfully utilizing nanotechnology under lab-scale experiments. This review critically evaluates the research outcomes on various nanomaterials used for the remediation of POPs from water bodies. The suggestions made after going through various pieces of literature are as follows: the surface properties of nanomaterials can be altered to the extent that they can be effectively utilized for the removal of POPs in a short period; second, more improvement can be made for the cost of the material (by using renewable/waste materials), reusability of material. More focus should be given to the green synthesis strategies as well as green nanotechnologies for environmental remediation, although more extensive studies need to be conducted to fill the knowledge gaps by doing a comparative analysis between the conventional and advanced treatment technologies. Further, the government and non-government organizations (NGOs) should make the general public aware of the potential dangers and consequences of POPs.

*Future Research:* In the future, more research is needed in the design and development of green and sustainable nanomaterials for the removal of POPs in an eco-friendly manner. This will lead to the further development of nanomaterials that can more effectively remove the range of POPs. Future research in the field must be focused on the treatment, absorption, and fate of the POPs and also on the interaction of nanomaterial with POPs. Scientists need to design advanced biobased nanomaterials for the removal of POPs in an environmentally friendly manner for a sustainable future, as well as making single technologies more efficient instead of going for a combination of two or more technologies. Most importantly, to forecast the sources, fate, and behaviors of POPs in the environment, researchers should focus more on the development of a risk-based screening model and framework in the future.

However, across the world, efforts are more focused on the complete ban of POPs instead of the removal of POPs.

## 8. Conclusions

In the present review, we have discussed the impacts, fate, and treatment strategies for persistent organic pollutants. POPs are present in very small concentrations that traditional technologies cannot sufficiently remove. There is a need to choose an advanced treatment based on the physicochemical characteristics of the pollutants. Nano-based technologies have revolutionized the area of wastewater treatment due to the unique properties of nanomaterials, which are ideal for various environmental applications. We have discussed the various nano-based processes, i.e., photocatalysis, nanoadsorption, and nanofiltration, for the effective removal of POPs. Though many nano-based removal technologies are still in the research stage, some have made their way to the pilot scale. To overcome the drawbacks, the collaboration between industry, research scientists, and governments is essential to provide a concrete solution to the challenges associated with the treatment of POPs.

## Figures and Tables

**Table 1:** List of POPs as per Stockholm Convention.

S. No	Chemical	Category
As per the 2001 Amendment (The Dirty Dozen)		
1	PCB	Industrial waste/byproduct
2	PCD	Byproduct
3	PCDF	Byproduct
4	Chlordane	Pesticide
5	Mirex	Pesticide
6	Endrin	Pesticide
7	Aldrin	Pesticide
8	Dieldrin	Pesticide
9	HCB	Pesticide
10	Heptachlor	Pesticide
11	Toxaphene	Pesticide
12	DDT	Pesticide
As per the 2009 Amendment		
13	Lindane	Pesticide
14	Chlordecone	Pesticide
15	Pentachloro benzene	Pesticide and Byproduct
16	Alpha-HCH	Pesticide and Byproduct
17	Beta-HCH	Pesticide and Byproduct
18	PFO and constituents PFOSF	Industrial
19	Hexabromobiphenyl	Industrial
20	Hexa-BDE and Hepta-BDE	Industrial
21	Tetra-BDE and Penta-BDE	Industrial
As per the 2011 Amendment		
22	Endosulfan	Pesticide

**Table 2:** Comparison between conventional and advanced nanotechnological processes.

Method	Technique	Advantage	Disadvantage
Chemical	Oxidation Reduction Hydrolysis Catalysis Photo-Fenton Ozonation Coagulation	Effective, rapid, and destructive use of hazardous chemicals	High cost, complex processes, toxic by-products, and end-products, sometimes the formation of even more toxic products than the parent pollutant
Physical	Adsorption Settling Membrane filtration Air stripping	Fast but comparatively less effective	The formation of by-products, cannot break the organic pollutant, high operational costs, low removal efficiency
Thermal	Combustion	Rapid and destructive, no by-product formed	High cost, complex process, not suggested for the recalcitrant compound
Biological	Microbial degradation Rhizoremediation Phytoremediation	Destructive, environmentally friendly, low energy requirement	Comparatively slow, high cost, less effective, toxic by-products/end-products, cost-benefit ratio is low
Nano-based	Photocatalysis Nanoadsorption Hybrid nanoremediation Nanofiltration	Superfast, high removal capacity, highly selective, highly efficient, excellent porosity, charge-based repulsion, comparatively less pressure, better selectivity	High cost, high operational cost and management, nanotoxicological concerns, membrane blocking, difficult to scale up

**Table 3:** Reviews in the field of POP removal using different nano-based processes.

S.NO	Technique	Nanomaterial Used	Reference
1.	Sulfate-radical-based advanced oxidation processes (SR-AOPs) for refractory organic contaminants	SR-AOPs using heterogeneous catalysis	[37]
2.	Hybrid photocatalytic membrane reactors for removal of POPs	Photocatalysis and membrane filtration	[38]
3.	Adsorptive and photocatalytic removal of POPs	Metal–organic frameworks (MOFs)	[39]
4.	Nanocatalysts and other nanomaterials for remediation of POPs	Oxidation, adsorption	[40]
5.	Photodegradation of POPs by GR-based composites	Catalysis and reduced graphene for photodegradation	[41]
6.	Nanoremediation for removal of POPs	Different nanomaterials, i.e., nanoscale zero-valent iron (nZVI), CNT, silica (SiO_2_), magnetic and metallic nanoparticles, graphene oxide, covalent organic frameworks (COFs), and MOFs	[42]
7.	Biogenic nanomaterials for the remediation of organic and inorganic pollutants	NMs, NPs, nanomembranes, and nanopowders for detection as well as for the removal of toxic metals and organic compounds	[43]
8.	Nanotechnology for pesticide removal from aqueous solutions	Nanomaterials, nanocomposites	[44]
9.	Removal of POPs using various multifunctional materials	Thermal, electrochemical, and photocatalytic remediation processes	[21]
10.	Remediation of water contaminated by poly- and perfluoroalkyl substances	Modified CNTs, modified nano-iron oxides, metal-based nanophotocatalysts	[45]
11.	Green synthesized nanoengineered materials for water/wastewater remediation	Green nanomaterials for POP removal	[46]
12.	Removal of persistent organic pollutants (POPs) from waters and wastewaters	Ionizing radiation, advanced oxidation and reduction processes (AO/RPs)	[47]
13.	Zinc oxide-based photocatalytic degradation of persistent pesticides	Photocatalytic degradation using ZnO	[48]
14.	Nanocatalysts and other nanomaterials for remediation of organic pollutants	Oxidation, adsorption, degrading organic pollutants for water remediation.	[40]
15.	Treatment of persistent organic pollutants in wastewater	Synergistic efficiency hydrodynamic cavitation with the advanced oxidation process	[49]
16.	Biofabricated nanoparticles for mitigating the environmental pollutants	Removal of pollutants via adsorption, immobilization, and reduction mechanisms	[50]
17.	Solid-phase microextraction of toxic pollutants using nanotechnology	Carbon-based materials, metal and metal-oxide nanomaterials	[51]
18.	Advanced nanotechnology and hybrid membrane-based treatment	Ag, Fe, Zn, Ti metal nanoparticles and carbon nanotubes	[52]
19.	Sustainable nanotechnology-based wastewater treatment	Graphene-based nanoparticles, their oxides (GO) and reduced graphene oxide (rGO), single-walled carbon nanotubes, multiple walled carbon nanotubes, covalent organic frameworks, metal, and metal-oxide-based nanoparticles	[53]
20.	Emerging contaminants removal from wastewater	Nanoscale materials such as nanosorbents, nanofilters, and nanocatalysts in the degradation of emerging contaminants	[54]
21.	Advanced oxidative processes for remediation of persistent organic pollutants from water	AOPs, such as sulfate radical, ionizing radiation, heterogeneous photocatalysis, electrohydraulic discharge system, ozonation, and Fenton processes	[55]
22.	Photocatalyst for organic-pollutant degradation	Carbon quantum-dot-supported zinc oxide (ZnO/CQDs)	[56]

**Table 4:** Nanomaterials are used for the removal of POPs.

Contaminant	Nanomaterials	References
Organic Pollutant	Ag/ZnO, ZnO-Bi, ZnO	[62]
	Mesoporous silica	[63]
	Graphene oxide-Ag NP	[64]
	TiO_2_	[65]
	ZnO	[66]
	TiO_2_-rGO	[67]
	Palladium and AgNp-embedded-zinc oxide nanostars	[68]
	RE^3+-^doped nano-TiO_2_	[69]
	MoS_2_/ZnS embedded in N/S doped carbon	[70]
	Magnetite and cobalt ferrite-decorated graphene oxide composite.	[71]
	CuO and NiO nanoparticles	[72]
Chlorpyrifos	ZnO/ZnO-Bi/ZnO-Ag/ZnO-Fe	[73]
	Potato-peel biochar	[74]
Aldrin	TiO_2_	[75]
Heptachlor	Fe/Cu nanoparticles	[76]
	Fe_2_O_3_	[77]
	NZVI	[78]
	Beta arsenene nanotubes	[79]
Mirex	Beta arsenene nanotubes	[79]
	Cu/Fe bimetal	[80]
Dimethoate	Gold Nanospheres and Nanorods	[81]
Chlordane	Graphene/Ni nanocomposite	[82]
	Cu/Fe bimetal	[80]
Endrin	Virgin (Fe_0_) and microbially regenerated (Fe_2+)_ iron	[83]
HCBs	Magnetic micro/nano FexOy-CeO_2_ composite	[84]
	Mg-doped Fe_3_O_4_	[85]
	Nano Pd (0)	[86]
	Nano zero-valent iron/activated carbon composite	[87]
	Co-Fe-O	[88]
	Zero-valent magnesium/graphite	[89]
	nZVI	[90]
PCBs	Nano Pd/Fe	[91]
	Zero Valent iron (ZVI)	[92]
	ZVI	[93]
	Pd nanocatalyst	[94]
	Ti-Ag Nanocomposite	[95]
	Fe_2_O_3_	[96]
	Au-Ag NP and Pd-Fe Bimetallic NP	[97]

## Data Availability

Not applicable.

## Abbreviations

AgNP	Silver Nanoparticles
EDs	Endocrine disruptors
EPA	Environment Protection Agency
FC	Food Chain
HCB	Hexachlorobenzene
MF	Microfiltration
NF	Nanofiltration
OCs	Organochlorines
OP	Organophosphorus
PAHs	Polyaromatic hydrocarbons
PCBs	Polychlorinated biphenyls
PCBzs	Polychlorinated Benzenes
PCDD	Polychlorinated dibenzo-p-dioxins
PCDF	Polychlorinated dibenzofurans
PCP	Personal Care Products
PDBE	Polybrominated diphenyl ethers
PF	Perfluorinated compounds
POPs	Persistent Organic Pollutants
RO	Reverse osmosis
ROS	Reactive Oxygen Species
SVR	Surface Volume Ratio
UF	Ultrafiltration
VOCs	Volatile Organic Compounds
ZnO	Zinc Oxide
